# Supplementary material for: Patterns of Intron Gain and Loss in Fungi
Source: PLoS Biol. 2004 Nov 30;2(12):e422. doi: 10.1371/journal.pbio.0020422 (PMC532390; doi:10.1371/journal.pbio.0020422)
Supplement: Table S1 — Also available at http://genes.mit.edu/NielsenEtAl/. (4.3 MB ZIP). [file pbio.0020422.st001.zip › NielsenEtAl/html/222.html]

AN5959.1.NCU00468.1.MG05276.1.FG04139.1


```
 CLUSTAL W (1.82) Multiple Sequence Alignments - Introns Inserted


Sequence 1: NCU00468.1	426 aa
Sequence 2: FG04139.1	472 aa
Sequence 3: MG05276.1	405 aa
Sequence 4: AN5959.1	367 aa
Alignment Length: 487 aa
Number Identitical Residues: 159 aa
Alignment Score (without introns) 8709


MG05276.1 	----------------~-----------MYARRLSAA~GWR2-------~------I~LA
NCU00468.1	MASTSESP--NMTGFT~VGLIGMGDMGKMYARRLSSA~---~-------~------V1VQ
FG04139.1 	MASHTQTVPDGMENFI~VGLIGMGDMGKMYAERLSAA~GWS2PSIFSSM0TLYLILL~VT
AN5959.1  	MITLILSATWFHYSLW2INACDKPDSFNNLKQEFEAY0S--~-------~-------~--
          	  :   : .    .    .  .  .  .   ..:.:  .                     

MG05276.1 	C~D----------------------------------~---QEEKYEALREEFSGHVC2-
NCU00468.1	M0N----------------------------------~-NIQILRNGHFVSRASDYII~Y
FG04139.1 	A~NPVPHAEWLTAVFYRILACDREENQESLQKEYAGN0KNIEICRNGHFVSRASDYII~Y
AN5959.1  	-~-----------------------------------~-GVTIYPNGHLVSRISDFIL~Y
          	                                       .        : .. *..:   

MG05276.1 	-VEAASIGRVVAQ~YGPS1TKQNAIVGGQTSCKDPEIKAFE~QHLPSDVDI0LLPGWTGV
NCU00468.1	SVEAAVIDRVVAQ~YGPS1TKLGAIVGGQTSCKDPEIKAFE~KYLPADVDI~VSCHSLHG
FG04139.1 	SVEAAVIDRVIAQ~YGPS1TKLGAIVGGQTSCKSPEIAAFE~THLPSDVDI~ISCHSLHG
AN5959.1  	SVEAGVIDKVVAD2----~--------------EPEGPAFD1SSLR-----~--------
          	:***. *.:*:*:                    .**  **:   *               

MG05276.1 	VCRDKLTWEDAQIQVLIPHRTNNSSSLATVEAILKCFNSTKVILTAREHDRITADTQAVT
NCU00468.1	PNVDPK----GQPLVLIKHRASDES-FSKVEHVLSCLNSKVVYLSADEHDRITADTQAVT
FG04139.1 	PNVDPY----NQPLVLIQHRAPDSA-LRKVETVLSCLKSKFVHLTAREHDRITADTQAVT
AN5959.1  	----------------------------FVEEVLSCFNSKYVYLSGEMHDRITADTQAVT
          	                             ** :*.*::*. * *:.  ************

MG05276.1 	HAAFLSMGKAWHASQEFPWEGARYVGGIENAKINLMLRIYAQKWHVYAGLAILNPEARKQ
NCU00468.1	HAAFLSMGKAWHATKQFPWEGTRYVGGIENVKINLMLRIYAQKWHVYAGLAILNPEAHKQ
FG04139.1 	HAAFLSMGKAWHANKQFPWELSRYVGGIENVKVNLMLRIYSQKWHVYAGLAILNPEAREQ
AN5959.1  	HAAFLSMGTAWQANKQFPWEISRWVGGIENVKINITLRIYSNKWHVYAGLAILNPAAKKQ
          	********.**:*.::**** :*:******.*:*: ****::************* *::*

MG05276.1 	IDQYARSTTELYKLMLEGRADELRKRVLAARDRVFGPEGAPKWEERPLLRDGLLDRFSLK
NCU00468.1	IAQFAKSTTELFQLMLEGHSDELRARVYAAKEKVFGAEGSPKWASKPLLRDDVLDQFSLR
FG04139.1 	ISQYARSVTDLYKLMLEGNREGFRERVYRARDKVFGP--STSWDTRPLLEPSILSSFSL-
AN5959.1  	IRQYAESVTELYKLMIEGKRDELKRRVRAAGAAVFRDG---TEKQDLLLSDEVLDRFSLS
          	* *:*.*.*:*::**:**. : :: **  *   **      .     **   :*. *** 

MG05276.1 	PEGSGTGERQQILPNNHLSLLAMVDCWSALGIVPFDHMICSTP~LFRLWLGVTEHLFRSP
NCU00468.1	QPENGGPSAP-SLPNNHLSLLAMVDCWSALGIVPYDHMICSTP~LFRLWLGVTEHLFRTP
FG04139.1 	----GTPTDE-PRPNNHLSILAMVDCWAALNIAPYDHMLCSTP~LFRLRLGVTEYLFRNG
AN5959.1  	---KGTRESSPPPPNNHLSLLAIVDCWSKLGIVPYDHMICSTP0LFRLWLGVTEYLFRNP
          	   .*        ******:**:****: *.*.*:***:**** **** *****:***. 

MG05276.1 	ARLDEALRIAVEDNTFRSDDLEFTFAAR~GWAECVSLGHFDTWRERFIVTQRFFEPRFAD
NCU00468.1	GLLDECLKVGIEDRTFRRDDLQFTIAAA~GWAECVGLRQFETWRERFAVTQAFFEPRFKG
FG04139.1 	DVLDAAIETAIDDKTYRSDDLEFTFAAR~AWAECVNLGHFETWEKRFVSTQEFFQPRFEE
AN5959.1  	DLLDEALDTAIDDKTFRSDDLEFTFAAR0AWSDCVSFGDFESYRDRFERIQQYFAPRFPD
          	  ** .:  .::*.*:* ***:**:**  .*::**.: .*:::..**   * :* ***  

MG05276.1 	AIKVGNEMVKAVLESSKD---
NCU00468.1	AVEVGNAMIKAVLASDEKD--
FG04139.1 	AKVVGNQMMKKILENSKDN--
AN5959.1  	AVKLGNEMMKRILEKTSNESS
          	*  :** *:* :* . ...::
```
